# Duns

**Published:** 1868-03-01

**Authors:** 


### *Duns.*

Nothing is more disagreeable than a dun; but it has this ameliorating circumstance, that it has a certain prophylactic — PREPAYMENT. Several thousand dollars of arrearages are shown upon the books of the JOURNAL. The only way to collect these is shown, by experience, to be by duns. The editor is willing to *give* his time and labor to the conduct of the JOURNAL, but finds it indispensable occasionally to call upon subscribers for dues. As the invitation to pay up is general, he trusts no one will consider the dun as a personal insult. And if any are omitted in passing the collector's hat, it is hoped they will not, it indebted to the JOURNAL, consider it as a personal slight. Duns are sent out by business agents—if mistakes occur in transaction of business matters with several thousand different persons, it should aggrieve no one. "Sharp" letters need not be penned, as all real mistakes will be most cheerfully rectified on simple notification.

